# Characterization and Comparative Overview of Complete Sequences of the First Plasmids of *Pandoraea* across Clinical and Non-clinical Strains

**DOI:** 10.3389/fmicb.2016.01606

**Published:** 2016-10-14

**Authors:** Delicia Yong, Kok Keng Tee, Wai-Fong Yin, Kok-Gan Chan

**Affiliations:** ^1^Department of Medical Microbiology, Faculty of Medicine, University of MalayaKuala Lumpur, Malaysia; ^2^Division of Genetics and Molecular Biology, Faculty of Science, Institute of Biological Sciences, University of MalayaKuala Lumpur, Malaysia

**Keywords:** conjugation, toxin-antitoxin systems, virulence genes, antibiotic resistance, plasmid

## Abstract

To date, information on plasmid analysis in *Pandoraea* spp. is scarce. To address the gap of knowledge on this, the complete sequences of eight plasmids from *Pandoraea* spp. namely *Pandoraea faecigallinarum* DSM 23572^T^ (pPF72-1, pPF72-2), *Pandoraea oxalativorans* DSM 23570^T^ (pPO70-1, pPO70-2, pPO70-3, pPO70-4), *Pandoraea vervacti* NS15 (pPV15) and *Pandoraea apista* DSM 16535^T^ (pPA35) were studied for the first time in this study. The information on plasmid sequences in *Pandoraea* spp. is useful as the sequences did not match any known plasmid sequence deposited in public databases. Replication genes were not identified in some plasmids, a situation that has led to the possibility of host interaction involvement. Some plasmids were also void of *par* genes and intriguingly, *repA* gene was also not discovered in these plasmids. This further leads to the hypothesis of host-plasmid interaction. Plasmid stabilization/stability protein-encoding genes were observed in some plasmids but were not established for participating in plasmid segregation. Toxin-antitoxin systems MazEF, VapBC, RelBE, YgiT-MqsR, HigBA, and ParDE were identified across the plasmids and their presence would improve plasmid maintenance. Conjugation genes were identified portraying the conjugation ability amongst *Pandoraea* plasmids. Additionally, we found a shared region amongst some of the plasmids that consists of conjugation genes. The identification of genes involved in replication, segregation, toxin-antitoxin systems and conjugation, would aid the design of drugs to prevent the survival or transmission of plasmids carrying pathogenic properties. Additionally, genes conferring virulence and antibiotic resistance were identified amongst the plasmids. The observed features in the plasmids shed light on the *Pandoraea* spp. as opportunistic pathogens.

## Introduction

Extrachromosomal DNA provides great impact in the evolution of bacteria in adapting to their surroundings. Depending on the genes it carries, it could act as a fitness factor to the host, or serve as a virulence factor by transforming the host into a pathogen. The impact is enhanced if coupled with the presence of functional genes for antibiotic resistance. Virulence factors enable bacteria to defend themselves against host defense systems whereas antibiotic resistance allows them to combat against antimicrobial treatment (Beceiro et al., 2013). Plasmids are one of the primary sources for extrachromosomal DNA in bacteria and are capable of self-transmission. Most importantly, plasmids were identified as leading factors to outbreaks such as the case for plasmid p1658/97 that contributed to β-lactam antibiotics resistance in *Escherichia coli* isolates during the clonal outbreak in a hospital in Warsaw, Poland (Zienkiewicz et al., 2007). In another situation, the outbreak of NDM-1-producing *Klebsiella pneumoniae* at the Kunning City Maternal and Child health hospital in China, may have been caused by the *bla*_NDM-1_-harboring plasmid that was present in all the clinical isolates (Zheng et al., 2016). Hence, the spread of virulence factors and antibiotic resistance properties caused by plasmids is possible, leading to a threat in public health.

*Pandoraea* (P.) genus consists of nine species namely *P. apista, P. pnomenusa, P. pulmonicola, P. sputorum, P. vervacti, P. oxalativorans, P. thiooxydans, P. norimbergensis*, and *P. faecigallinarum* (Coenye et al., 2000; Anandham et al., 2010; Sahin et al., 2011) with rod-shape, aerobic and gram-negative properties. Non-clinical strains have been isolated from soil, chicken dung and oxic water layer (Coenye et al., 2000; Anandham et al., 2010; Sahin et al., 2011). Little is known about the pathogenic potential of these strains. Clinical strains have been isolated from cystic fibrosis (CF) patients (Coenye et al., 2000) as well as non-CF patients (Stryjewski et al., 2003; Pimentel and MacLeod, 2008). There is very limited information on the factors contributing to the pathogenicity potential in *Pandoraea*. It is also not known if the virulence of *Pandoraea* is linked to the existence of plasmids. Previous studies have reported on the contribution of plasmids to the virulence of members of *Burkholderia cepacia* complex (BCC) (Agnoli et al., 2014) and *Mycobacterium avium-intracellulare* (MAI) complex (Hellyer et al., 1991). Similar to *Pandoraea*, both these bacterial groups are opportunistic pathogens (Mahenthiralingam et al., 2008; Whiley et al., 2015). This leads to a speculation that the pathogenicity of *Pandoraea* could be contributed by the presence of plasmids.

Four completely sequenced *Pandoraea* strains harbored eight plasmids in total. Along with five previously reported plasmids from *P. oxalativorans* DSM 23570^T^ (pPO70-1, pPO70-2, pPO70-3, pPO70-4) (Chan et al., 2016) and *P. vervacti* NS15 (pPV15) (Ee et al., 2015), three novel plasmids were sequenced for comparison in this study. Here, we present the first comparative study of plasmids within the *Pandoraea* genus. Our aims are to annotate and study their properties for replication, segregation, conjugation and presence of toxin-antitoxin (TA) systems. These systems have been reported responsible in maintaining the survival and replication of plasmids (Thomas, 2000; Kroll et al., 2010; Baxter and Funnell, 2014). Knowledge on these systems is vital in disease treatment in which drug design can be employed (Sengupta and Austin, 2011). Additionally, we also narrowed our search for virulence and antibiotic resistance characteristics within the plasmids. The identification and study of plasmids are important to discover the features of novel plasmids, especially in opportunistic pathogens such as *Pandoraea*. These sequence data analyses will hopefully serve as a basis for future research.

## Materials and methods

### Bacterial strains, plasmids, culture conditions, and sequencing

The *Pandoraea* species isolates *P. faecigallinarum* DSM 23572^T^ and *P. apista* DSM 16535^T^ were obtained from the Leibniz Institute DSMZ—German Collection of Microorganisms and Cell Cultures GmbH. The isolates were cultured on Luria-Bertani medium for 24 h at 28 °C with shaking at 220 rpm in reference to a previous protocol (Lim et al., 2014). Genomic DNA was isolated using an Epicentre Masterpure DNA extraction kit (Epicentre, Inc., Madison, WI, USA) according to the manufacturer's instructions. To obtain complete genome sequences for a better genomic overview, Pacific Biosciences (PacBio) RS II Single Molecule Real Time (SMRT) sequencing method (Pacific Biosciences, Menlo Park, CA) was employed. Genomic DNA was processed into SMRTbell™ library using the SMRTbell template preparation kit 1.0 according to the “Procedure and checklist-20 kb template preparation using BluePippin size-selection system” protocol as reported by Lim et al. (2016). Sequencing of the SMRTbell™ libraries were conducted using P6 DNA polymerase (C4 chemistry) which is PacBio's latest DNA sequencing chemistry that improves the average read length (http://investor.pacificbiosciences.com/releasedetail.cfm?releaseid=876252). Quality filtering and *de novo* assembly of the sequenced reads were performed using the Hierarchical Genome Assembly Process (HGAP) version 2 of PacBio (DevNet; Pacific Biosciences).

Based on the PacBio Circularizing and Trimming wiki at (https://github.com/PacificBiosciences/Bioinformatics-Training/wiki/Circularizing-and-trimming), circularity of all contigs generated from the assembly was determined based on reads mapping across the beginning and end of the contigs. To confirm this, Gepard (Krumsiek et al., 2007) was used to generate dot plots for each contig to detect the presence of self-similar region at the 5′ and 3′ contig ends. The contigs were then subjected to PacBio Quiver algorithm for polishing prior to further analysis. Chromosomal contigs were determined based on the similarity of the contig sizes to the expected chromosomal size of *Pandoraea* and were further confirmed by using BLASTN. Subsequently, all extra-chromosomal contigs were aligned against the chromosomal contigs and were assessed using Contiguity (Sullivan et al., 2015). Only contigs which demonstrated low or no similarity to their respective chromosomal contigs were determined as plasmid contigs.

As a result, three plasmid contigs were observed. Non-clinical isolate *P. faecigallinarum* DSM 23572^T^ harbors two plasmids (pPF72-1, pPF72-2) whereas clinical isolate *P. apista* DSM 16535^T^ carries a single plasmid (pPA35). Complete sequences of plasmids from non-clinical strains *P. oxalativorans* DSM 23570^T^ (pPO70-1, pPO70-2, pPO70-3, pPO70-4) (Chan et al., 2016) and *P. vervacti* NS15 (pPV15) (Ee et al., 2015) were obtained from NCBI.

### Bioinformatics analysis

Prokaryotic Dynamic Programming Genefinding Algorithm (Prodigal) Version 2.6.2 (Hyatt et al., 2010) was used in the prediction of open reading frames. The predicted sequences were then functionally annotated by performing homology search against the NCBI nr database, InterProScan (Quevillon et al., 2005) and the Conserved Domain Database (CDD) (Marchler-Bauer et al., 2015). Identification of key genes conferring biological advantages were performed based on Virulence Factors Database (VFDB) (Chen et al., 2005), The Comprehensive Antibiotic Resistance Database (CARD) (McArthur et al., 2013) and TAfinder (http://202.120.12.133/TAfinder/index.php).

### Nucleotide sequence accession numbers

The accession numbers of the complete sequences of the three plasmids sequenced in this study and of the five plasmids from NCBI can be found in GenBank with the accession numbers CP011808 (pPF72-1), CP011809 (pPF72-2), CP013482 (pPA35), CP011518 (pPO70-1), CP011519 (pPO70-2), CP011520 (pPO70-3), CP011521 (pPO70-4), and CP010898 (pPV15).

## Results and discussion

### General features of plasmids

All eight plasmids range in sizes from 46,278 to 640,227 bp, organized in a single contig with overall G + C content in the range of 57.81–63.8%. The number of open reading frames in the range of 65–626 was predicted across the plasmids in which 62–591 are protein-encoding genes (Supplementary Figures S1–S4 and Supplementary Table S1). The features of each plasmid are shown in Table 1. The putative origin of vegetative replication (*oriV*) and terminus (*terV*) for each plasmid were identified based on cumulative GC-skew analysis using GenSkew (http://genskew.csb.univie.ac.at/) (Supplementary Figures S5–S8). The predicted oriV and terV are marked by the minimum and maximum values of the cumulative GC-skew, respectively, which corresponds to the nucleotide positions (Grigoriev, 1998). Overall, *Pandoraea* plasmid sequences yielded low similarity against plasmid sequences from the RefSeq and EMBL nucleotide sequence database with sequence completeness of <12%. In addition, no homologous gene organization was observed. Hence, no comparison between regions of the *Pandoraea* plasmids and other known plasmids could be made. These findings indicate that the *Pandoraea* plasmid sequences were not reported before.

**Table 1:** **General plasmid features**.

**Plasmid name**	**Host strain**	**Isolation source**	**Size (bp)**	**No. of open reading frames**	**No. of protein-encoding genes**	**G + C content (%)**	**Accession no**.
pPF72-1	*P. faecigallinarum* DSM 23572^T^	Chicken dung	402292	444	434	61	CP011808
pPF72-2	*P. faecigallinarum* DSM 23572^T^	Chicken dung	124395	155	154	59.3	CP011809
pPA35	*P. apista* DSM 16535^T^	Sputum of CF patient	77293	107	105	57.81	CP013482
pPO70-1^a^	*P. oxalativorans* DSM 23570^T^	Oxic water layer	640227	626	591	63.8	CP011518
pPO70-2^a^	*P. oxalativorans* DSM 23570^T^	Oxic water layer	135985	154	153	60.6	CP011519
pPO70-3^a^	*P. oxalativorans* DSM 23570^T^	Oxic water layer	85789	120	116	59.8	CP011520
pPO70-4^a^	*P. oxalativorans* DSM 23570^T^	Oxic water layer	46278	65	62	59.2	CP011521
pPV15^b^	*P. vervacti* NS15	Soil	105231	118	115	62	CP010898

a *From previously published data of P. oxalativoranss DSM 23570^T^ genome (Chan et al., 2016)*.

b *From previously published data of P. vervacti NS15 genome (Ee et al., 2015)*.

To determine the possible origins of genes within the *Pandoraea* plasmids, we performed a search against a non-plasmids database. The database was constructed based on bacterial genome sequences obtained from NCBI. We observed that majority of the genes across the plasmids matched to genomes of *Burkholderia, Bordetella, Xanthomonas, Ralstonia, Pseudomonas, Alicycliphilus, Nitrosomonas* and *Cupriavidus* with similarity and completeness scores well above 50%.

### Origin of vegetative replication, terminus, and replication genes

Plasmid replication is vital in ensuring reproducibility and segregation of plasmids into daughter cells during host cell division. This is an essential aspect in the plasmid maintenance system and highlights the values of identifying the potential genes involved in replication machinery, especially in the unknown potential of plasmids in *Pandoraea*.

Putative replication initiator protein-encoding gene (*repA*) was identified in pPO70-4 and pPA35, indicating self-replication potential of these two plasmids. However, no *repA* was identified in the other plasmids. This has led us to speculate that a different replication mechanism exists in which a plasmid initiator protein is not employed, such as the case for plasmid ColE1 (del Solar et al., 1998) that reportedly uses DNA polymerase I for replication initiation.

Putative genes surrounding the putative predicted *oriV* and *terV* locations that could be involved in replication were identified. In pPA35, *oriV* was identified at position 72,001 bp within an acyltransferase gene whereas *terV* was identified at position 31,001 bp within an intergenic region between conjugal transfer TraM containing domain protein and hypothetical protein-encoding genes. Conjugation *tra* and *trb* genes were found to flank either side of the *terV*. A similar observation was made in plasmid pRF in which its *terV* was flanked by conjugation genes (Gillespie et al., 2007). Other genes located downstream of the *terV* in pPA35 are partitioning genes (*parA, parB*) and *repA*. A single-strand DNA-binding (SSB) protein-encoding gene (*ssb*) was found located next to *repA* and could be associated with DNA replication (Meyer and Laine, 1990).

The *oriV* at position 37,001 bp in pPO70-4 was found within an antitoxin gene. Located upstream are genes *repA, ssb* and a gene encoding for a partitioning protein which shares 96% identity with ParA of *Xanthomonas*. Another partitioning protein with a ParA domain was found downstream of the *oriV* and has 99% identity to the upstream partitioning protein-encoding gene. At position 25,001 bp, the *terV* is located within a type IV secretion system (T4SS) protein-encoding gene flanked on either side by conjugation genes. This is another feature similar to plasmid pRF (Gillespie et al., 2007).

As for pPF72-1, the *oriV* at position 250,000 bp is found within an intergenic region between *parA* and a hypothetical protein-encoding gene, a situation that matches the finding that *parA* is often located adjacent to *oriV* (Picardeau et al., 2000). The *terV* at position 332,001 bp is located within a hypothetical protein-encoding gene. Plasmid pPF72-2 on the other hand, has its *oriV* at position 124,001 bp in an intergenic region between transposase and hypothetical protein-encoding genes whereas its *terV* at 30,001 bp is found within a hypothetical protein-encoding gene with *ssb* located downstream.

Overall, only pPA35, pPF72-1, pPF72-2 and pPO70-4 showed the presence of genes that are possibly involved in their replication systems. Plasmids pPO70-1, pPO70-2, pPO70-3 and pPV15 showed no signs of replication genes located near the *oriV* and *terV* as well as in other parts of the plasmid. The observed characteristics raised the hypothesis of plasmid-host interaction for plasmid replication, as reported for plasmid Pps10 from *Pseudomonas syringae* (Fernández-Tresguerres et al., 1995). The absence of replication genes in the four plasmids motivates the understanding of the interaction between the plasmids and their respective host strains. The information can be potentially used to design the plasmids with a host-killing system, and enable them to be released into the environment as an effort to control possible infection by *Pandoraea* and other opportunistic pathogens.

### Partitioning genes

Partitioning genes function in the segregation of plasmids into daughter cells following plasmid replication. The segregation activity, that takes place as part of the second mechanism of plasmid maintenance, is also known as the partition system or *par* system. In the identification of putative partitioning genes, genes *parA* and *parB* of the type I *par* system (Gerdes et al., 2000) were observed in pPF72-1 and pPA35. These genes are *trans*-acting protein-encoding genes whereby *parA* encodes an ATPase that regulates the function of DNA binding protein-encoding *parB* (Bignell and Thomas, 2001). Besides the two aforementioned plasmids, pPO70-4 was found to contain two partitioning protein-encoding genes with a ParA domain identified in each gene.

No *par* genes were identified in pPF72-2, pPO70-1, pPO70-2, pPO70-3 and pPV15, indicating a possibly different mode of segregation besides the typical partition system. This situation is possible considering previous studies conducted on plasmids that describe the usage of other approaches for segregation other than the *par* system. One of these studies involves plasmid pSK1 from *Staphylococcus aureus*, in which an identified gene is involved in the increase of segregation stability while no ATPase activity was required to drive plasmid segregation (Simpson et al., 2003). In another study, plasmid R388 utilizes other genes instead of the *par* system for plasmid segregation (Guynet et al., 2011).

Interestingly, all the *Pandoraea* plasmids without *par* genes also did not contain *repA*. The consistent observations on this absence of replication and partitioning genes, strengthen the view on close interactions between the host strains and plasmids for the maintenance system. Plasmid stabilization/stability protein-encoding genes have been observed across pPF72-1, pPF72-2, pPO70-2, pPO70-3 and pPV15, possibly playing a part in plasmid segregation but this has not been confirmed.

### Toxin-antitoxin systems

Toxin-antitoxin (TA) systems in plasmids form the third mechanism of plasmid maintenance. They have also been termed as post-segregational killing systems or plasmid addiction systems (Kroll et al., 2010). A single TA system consists of an antitoxin and a toxin protein. In the event that a daughter cell inherits a plasmid, the antitoxin protein acts to neutralize the action of the toxin, thereby preventing cell death (Sengupta and Austin, 2011). However, when plasmids are not distributed to daughter cells, the stable toxin protein causes cell death whereas the unstable antitoxin protein is degraded. In other words, TA systems prolong the presence of plasmids in the bacterial population by eliminating plasmid-free cells that have arisen due to unsuccessful plasmid replication and segregation. There are five classes of TA systems of which the class Type II is the best studied (Unterholzner et al., 2013). Hence, we have focused only on class Type II TA systems in our study.

The presence of putative genes known to behave as TA systems, have been identified across the *Pandoraea* plasmids except for pPV15. Supplementary Table S2 displays the list of TA systems found in each of the plasmids.

More than one TA system was observed in each of the plasmids except in pPO70-4. The observed presence of TA systems could be due to the nature of TA as a mobile element, and is subsequently integrated into the DNA of the plasmids. The presence of TA systems would enhance the post-segregational activity, and in turn ensure better plasmid maintenance. With the exception of pPO70-4, all plasmids in free-living strains have more TA systems as compared to pPA35, which originates from a host-associated strain. A study carried out by Pandey and Gerdes (2005) presented that free-living organisms possess multiple TA systems as compared to host-associated organisms that did not have any. This observation is similar to the *Pandoraea* plasmids from free-living strains, whereby the presence of TA systems proposes the need for survival in the constantly changing ecosystem. However, the identification of two TA systems in pPA35, suggests that the maintenance of this host-associated strain-harboring plasmid is still essential in adapting to an inconsistent environment.

A total of seven types of TA systems were observed across the *Pandoraea* plasmids namely MazEF, VapBC, RelBE, YgiT-MqsR, HigBA and ParDE. The TA systems VapBC and RelBE of pPF72-1 and pPO70-3, respectively, were found within plasmid stabilization protein-encoding genes. It is proposed that these TA systems are helpful in preventing the loss of these elements in daughter cells. All plasmids except pPO70-2 and pPO70-4, have more than one copy of some of its TA systems. Such a situation will ensure a stable single-copy plasmid inheritance (Venkatesan et al., 2001). All TA systems identified in the *Pandoraea* plasmids were encoded near transposase or integrase encoding genes, indicating an ability to be mobilized by mobile genetic elements (Bustamante et al., 2014).

The orientation of type II TA systems is such that the antitoxin gene is situated upstream of the toxin gene (Van Melderen and Saavedra De Bast, 2009). However, we observed that some TA systems in the *Pandoraea* plasmids have an opposite orientation. This was observed amongst MazEF, VapBC, RelBE, HigBA and HipBA across pPF72-1, pPF72-2, pPO70-1 and pPO70-3, whereby RelBE appears to be the most frequent in number having an opposite orientation. Previous studies have also shown the same unusual occurrence in TA system orientation (Budde et al., 2007; Yamaguchi et al., 2009; Christensen-Dalsgaard et al., 2010).

Gene overlap in the TA systems has been observed across the *Pandoraea* plasmids. The occurrence of gene overlap in prokaryotes indicates translational coupling and this phenomenon is common in TA systems (Pandey and Gerdes, 2005). An overlap of 4 and 1 bp in TA systems were observed in pPF72-1, pPF72-2, pPO70-1, pPO70-2, pPO70-4 and pPA35. Generally, most TA systems have an overlap of 4 bp or even 1 bp, in which the antitoxin stop codon overlaps the start codon of the toxin gene (Pandey and Gerdes, 2005). Other overlap sizes have also been observed amongst the *Pandoraea* plasmids. Overlaps of 7 and 5 bp were found within MazEF and RelBE, respectively, in pPF72-1. Plasmid pPF72-2 has 29, 11, and 20 bp overlaps in its RelBE whereas a 9 bp overlap is seen in HipBA in pPO70-1. Lastly, 13 and 29 bp overlaps were identified in RelBE in pPO70-3. Chan et al. (2012) have previously reported on other overlap sizes besides the usual 4 and 1 bp overlaps. Some TA systems in the *Pandoraea* plasmids have no gene overlap such as in pPF72-1, in which gaps of 118 bp (MazFike_domain, higA) and 47 bp (RelBE) were observed whereas a gap of 170 bp was identified within RelBE in pPO70-3.

### Conjugation

Conjugation is one of the mechanisms of bacterial lateral gene transfer (Burrus and Waldor, 2004). Genes enabling conjugation are often located on plasmids and genetic transfer occurs when these conjugative plasmids are self-transmitted during bacterial cell to cell contact. The mode of conjugation is important as it enables plasmids to deliver features that can be beneficial to the host strains. An example has been reported by Rösch et al. (2014) where the presence of conjugative plasmid pLS20 conferred stress resistance to its host strain. The identification of the conjugation mechanism can also be helpful in preventing the transfer of pathogenic properties such as reported by Lujan et al. (2007), who described the disruption of relaxase to prevent antibiotic resistance transfer. The conjugative potential of *Pandoraea* plasmids has been predicted based on the presence of putative conjugation genes encoding T4SS components. Overall, these genes were predicted as *tra, trb, trw* and *virB*. These different gene designations exist overtime for different plasmids in particularly when they are categorized into different incompatibility groups (Stolz, 2014). Some of the examples of this categorization include *tra, trb* and *trw* of incompatibility groups IncF and IncN, IncP and IncW, respectively, as well as *virB* of Ti plasmids (Christie, 1997). All these gene designations have been identified in the conjugation genes among the *Pandoraea* plasmids. No conjugation genes however, were identified in pPO70-3.

Conjugation genes in pPA35 and pPO70-4 were predicted as *tra, trb* and *virB*. In pPA35, two separate regions exist mainly coding for *tra* (*tra* region) and *trb (trb* region) genes, respectively. Similar regions were observed in plasmid pTiC58 of *Agrobacterium tumefaciens* (Alt-Mörbe et al., 1996). The *tra* region contains genes *traC, traG, traJ* and *traL*, in which all except *traL* are located on the lagging strand. Genes encoding a relaxase and peptidase S26, were also identified in this region in which the latter contains conjugal transfer peptidase TraF domain. An additional gene adjacent to the *tra* region was characterized with conjugal transfer protein TraM domain and is located on the leading strand. Genes in the *trb* region are *trbB, trbC, trbD, trbG, trbI, trbJ, trbL* and *traX*. A single *virB4* has also been identified in this region. *Five* additional genes were observed within and adjacent to the *trb* region with domains TrbA, TrbF, TrbH, TrbM and TrbN, respectively. For the *trb* region, the conjugation and additional genes were located on the leading strand except for the additional gene with conjugal transfer protein TrbA domain.

In contrast to the two conjugative regions in pPA35, pPO70-4 comprises a single conjugative region which contains genes *traC, trbL, traB, virB10* and *virB3*. Besides these, genes encoding pilus assembly protein CpaF and T4SS protein were also identified. Three additional genes were identified within and adjacent to the conjugative region of pPO70-4. These genes have domains TrbG, TrbF and T4SS lytic transglycosylase VirB1, respectively. Conjugation genes in pPO70-4 are all located on the lagging strand except for *traC*.

The best hit was identified for each additional gene via homology search with BLASTP against the nr database. Information on their domain and best hits are tabulated in Supplementary Table S3. They were found to carry conjugation domains and have close hits to conjugation genes suggesting that they are yet to be reported as conjugative-related genes.

In comparison to the conjugation genes in pPA35 and pPO70-4, the other *Pandoraea* plasmids contain differently annotated conjugation genes, except for a single Tra coupling protein-encoding gene (*traD*) found in pPO70-1, pPO70-2 and pPV15. These genes include *trwB, virB11* and *virB9*. Relaxase, endonuclease and lytic transglycosylase and type VI secretion system (T6SS) protein-encoding genes were also identified.

Domain search revealed a T6SS lytic transglycosylase VirB1 domain within the lytic transglycosylase encoding genes, indicating that they could function in degrading the peptidoglycan wall in order to permit construction of structures such as the pilus during conjugation (Wallden et al., 2010). Furthermore, these genes have 59-65% identity with T4SS protein VirB1 of *Xanthomonas fuscans* subsp. *Aurantifolii* str. ICPB 11122. According to Wang and Macrina (1995), the endonuclease, also known as the nickase, functions in carrying out a nick at the *oriT* during conjugation. It is suggestive that the endonuclease gene identified in the *Pandoraea* plasmids, may act in a similar way. The endonuclease genes found in pPF72-2, pPO70-1 and pPV15, share similarity to the plasmid conjugative transfer endonuclease encoding gene of *Collimonas arenae* (60% identity), *Burkholderia* sp. AU4i (69% identity) and *Burkholderia cenocepacia* H111 (69% identity), respectively.

**Figure 1:**
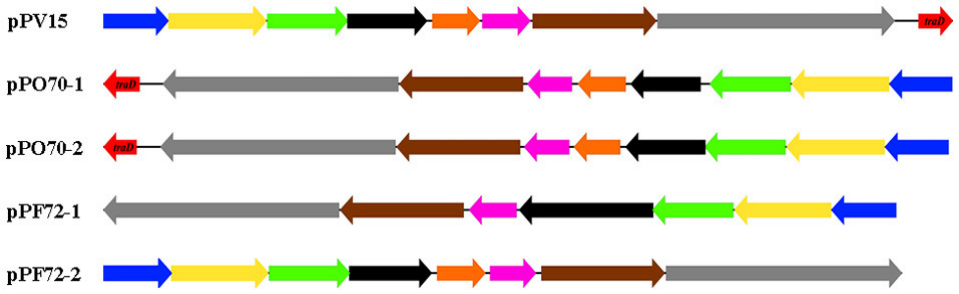
**Genetic arrangement of the shared conjugation region of plasmids pPF72-1, pPF2-2, pPO70-1, pPO70-2 and pPV15**. Predicted conjugation genes and their orientation are presented in block arrows. The different genes are represented in colors: *virB9* (blue), type VI secretion system (T6SS) protein-encoding gene (yellow), *virB11* (green), lytic transglycosylase protein encoding gene (black), hypothetical protein encoding gene (orange), endonuclease protein encoding gene (pink), *trwB* (brown), relaxase protein encoding gene (gray), *traD* (red). Difference in orientation of the conjugation regions amongst the plasmids are represented by the direction of the block arrows. Block arrows pointing toward the left indicate reverse strand orientation whereas block arrows pointing toward the right indicate forward strand orientation.

The conjugation region in pPF72-1, pPF72-2, pPO70-1, pPO70-2 and pPV15 is presented in Figure 1 as the only significant region that is shared amongst the *Pandoraea* plasmids. The shared region contains the same conjugation genes except for *traD*, which is only present in pPO70-1, pPO70-2 and pPV15. Gene arrangements in this region appear to be conserved amongst the five plasmids. Pairwise comparisons between the conjugation regions yielded 88–100% nucleotide identity values shared between the respective genes, further leading to the possibility of common origin for these regions.

### Other genes

#### Virulence and antibiotic resistance genes

One of the important features of plasmids is the capability of carrying genes conferring virulence and antibiotic resistance properties, and transmitting these properties to new host strains. To explore the virulence and antibiotic resistance properties delivered by *Pandoraea* plasmids, we identified putative virulence and antibiotic resistance genes based on the search against VFDB (Chen et al., 2005) (Supplementary Table S4) and CARD (McArthur et al., 2013) (Supplementary Table S5), respectively, employing a cutoff value of 40% identity and 50% coverage.

All eight *Pandoraea* plasmids were found to carry putative virulence genes with a total ranging from 3 to 91. The genes mainly encode functions related to type II (T2SS), III (T3SS), and IV (T4SS) secretion systems, purine biosynthesis, iron acquisition, DNA repair, replication and recombination, adherence, immune evasion, surface protein, motility, ureolytic and sialidase activities.

Plasmid pPA35 carries virulence genes encoding functions involved in T4SS (*pilD, lepB*). A previous study by Paranjpye et al. (1998), showed that mutation in *pilD* caused a defect in pilus expression and protein transport using the type II secretion pathway. This has led to a reduced adherence to epithelial tissue culture, cytotoxic activity and virulence. The *lepB* gene, encoding a SPAase homolog, is known to be necessary for viability (Waite et al., 2012). A *purC* gene encoding phosphoribosylaminoimidazole-succinocarboxamide synthetase involved in purine biosynthesis, has also been predicted in pPA35. Mutation in this gene was found to minimize cytotoxicity and formation of biofilm in the infection-causing bacterium, *Pseudomonas aeruginosa*, among CF patients (Guo et al., 2016). In addition, Jackson et al. (1999) presented that mutation of *purC* affected the growth ability of *M. tuberculosis* and *M. bovis* BCG in mouse bone marrow macrophages.

Compared to the number of virulence genes observed in pPA35, the plasmids of non-clinical host strains harbor more virulence genes, which will enable the respective host strains to adapt to their constantly changing environment. Besides that, in the event that these host strains are transmitted into a clinical setting, they may confer pathogenicity. Majority of the virulence genes in plasmids from non-clinical strains encode transposases, indicating the means of adaptive evolution of the plasmids. Other virulence genes that were mostly observed as well, are those which encode functions related to T3SS and T4SS.

Plasmid pPF72-1 carries virulence genes encoding functions related to T3SS (*ssph2, ssaJ, escN*), T4SS (*pilB*), motility (*flgM*), iron acquisition (*entD, hxuB*) and cell surface (*bca*). Virulence gene *ssph2* is a *Salmonella typhimurium* gene, that has participated in causing virulence in calves (Miao et al., 1999) whereas *ssaJ*, which encodes a T3SS component of *Salmonella* pathogenicity island (SPI-2), was found to possibly lead to reduction of virulence upon a transposon insertion in the gene (Hensel et al., 1997). The *escN* gene on the other hand encodes the T3SS ATPase of *Escherichia coli* (Andrade et al., 2007), which together with proton motive force, provides energy for secretion (Minamino and Namba, 2008). Assembly ATPase of the T4SS pilus encoded by *pilB*, functions in giving rise to virulence upon its encounter with host tissues (Persat et al., 2015). Gene *flgM*, which encodes a negative regulator of flagellin synthesis, causes depletion of *S. typhimurium* in a typhoid fever mouse model upon mutation (Schmitt et al., 1994). Iron acquisition gene *entD* resulted in virulence termination of extra-intestinal pathogenic *E. coli* (ExPEC) upon inactivation (Martin et al., 2013) whereas *hxuB* is part of the haem-haemopexin utilization gene cluster with a role in virulence (Morton et al., 2007). Lastly, *bca*, which encodes for C protein alpha-antigen, causes a decrease in bacterial virulence upon knock-out mutation and is significant in group B Streptococcus pathogenicity (Li et al., 1997).

Genes encoding functions related to T4SS components (*trwD, BMEII0028*—encodes a VirB4 ATPase homolog) (Vayssier-Taussat et al., 2010; Wallden et al., 2010)and DNA repair, replication and recombination (*ssb*), were identified in pPF72-2. The *ssb* gene encodes SSB, which controls the activity of RecBCD nuclease as reported in *E. coli* (Anderson and Kowalczykowski, 1998). Loss of function of this nuclease will lead to a decrease in virulence as observed in *S. enterica* (Cano et al., 2002).

Only T3SS component encoding genes (*ssaT, bscj, escS*) (Hensel et al., 1997 and Tomich et al., 2003) have been identified in pPO70-1. A previous study by Deiwick et al. (1998) showed that mutation in *ssaT* caused a decrease in gene expression of *S. typhimurium* pathogenicity island, SP1. Additionally, sensitivity to gentamicin and polymyxin B antibiotics also resulted from the mutation. The protein encoded by *bscj* forms the T3SS channel, which functions to secrete effector proteins (Plano et al., 2001). Virulence gene *escS* on the other hand, encodes the T3SS component of enteropathogenic *E. coli*. Besides that, several putative genes encoding proteins involved in the production of urease and its activation were discovered in pPO70-1. These genes encode urease subunits alpha, beta and gamma, as well as urease accessory proteins UreD, UreE, UreF, and UreG, which are all involved ureolytic activity (Koper et al., 2004). Products of ureolytic activity are exploited to maintain surrounding pH (Burton and Prosser, 2001). The presence of these genes will enable the host strain to adapt in a urea-rich environment. Besides that, the production of urease also contributes as a virulence factor among human pathogens such as *Helicobacter pylori*, as well as in fungi namely *Cryptococcus neoformans* and *Coccidioides posadasii* (Cox et al., 2000; Rutherford, 2014).

The T4SS component encoding gene *BMEII0025* (encodes an attachment mediating protein VirB1 homolog) (Wallden et al., 2010) has been identified in pPO70-2 along with an adherence gene (*efa1*) and two sialidase (*nanj*) genes. Gene *efa1* was identified in enterohaemorrhagic *E. coli* which was shown to play a part in cell adherence (Nicholls et al., 2000). Chiarezza et al. (2009) have described *nanj* to have effect in increasing alpha-toxin-mediated cytotoxicity, however is not essential for virulence relating to gas gangrene.

Two T4SS related genes (*bepF*) have been identified in pPO70-3 encoding a *Bartonella* (*B*.) effector protein, which in a previous study was translocated via *B. henselae T4SS* into infected endothelial cells (Schmid et al., 2004).

As compared to pPO70-1, pPO70-2 and pPO70-3, only pPO70-4 was found to contain a virulence factor encoding gene related toT2SS (*gspD*). The T2SS, also known as the general secretory pathway (GSP), has been previously reported to be involved in bacterial virulence (Iwobi et al., 2003; Baldi et al., 2012). The *gspD* gene encodes a protein that forms the pore of T2SS in the outer membrane. Virulence gene *pcrV* was also identified in pPO70-4, encoding an important component in *Pseudomonas* T3SS which plays a part in cytotoxicity (Nanao et al., 2003). Besides that, genes encoding components of T4SS (*trwD, trwM, BMEII0025—*encodes an attachment mediating protein VirB1 homolog, *BMEII0034*—encodes a channel protein *VirB10* homolog) have also been identified in pPO70-4 (Vayssier-Taussat et al., 2010; Wallden et al., 2010) along with *tviB* which encodes an enzyme contributing to virulence antigen Vi formation in *S. typhi* (Zhang et al., 2006).

The adherence gene *fnbB* identified in pPV15, encodes fibronectin-binding protein B which was shown to be present in majority of *S. aureus* isolates from different infection types, indicating a contribution to virulence (Arciola et al., 2005).

Antibiotic resistance genes were predicted in all *Pandoraea* plasmids except in pPA35 and pPO70-3. These genes were predicted at a small number ranging from 1 to 6 with pPF72-2 and pPO70-2 having the least. The predicted genes confer resistance to tetracyclin, beta-lactamase, aminoglycoside, fluoroquinolone, chloramphenicol, lincosamide, and macrolide resistance.

Genes encoding multidrug ABC transporter ATPases were observed in plasmids pPF72-1, pPO70-1 and pPV15. This subunit, which forms part of the ABC transporter, functions in promoting the transport of cytotoxic drugs across the membrane. Tetracycline resistance genes *tetM* and *tet36* have been identified in pPV15 and pPO70-1, respectively, where both encode ribosomal protection proteins (Whittle et al., 2003; Dönhöfer et al., 2012). Genes encoding proteins involved in beta-lactamase resistance, FEZ-1 beta-lactamase (*blaFEZ-1*) (Mercuri et al., 2001) and AQU-1-type AmpC beta-lactamase (Pérez-Pérez and Hanson, 2002), were identified in pPO70-1 and pPO70-4, respectively. On the other hand, genes conferring resistance to aminoglycoside (*rph*) (Shaw et al., 1993) and fluoroquinolone (*gyrB*) (Nasri Yaiche et al., 2014), were found in pPF72-2 and pPO70-1, respectively. Additionally, genes involved in macrolide (*mgt*) (Quirós et al., 2000) and chloramphenicol (*cmlA4*) (Poirel et al., 2000) resistance were also identified in pPO70-1. Lincosamide resistance gene (*lnuB*) (Almuzara et al., 2013) was found in pPO70-2 whereas pPO70-4 contains two genes (*mel*) that are associated with macrolide resistance (Ambrose et al., 2005).

It is observed that antibiotic resistance genes were detected only in plasmids from non-clinical *Pandoraea* strains which have not been isolated previously from any clinical source. We hypothesize that these plasmids convey the respective host strains with antibiotic resistance properties that are essential to survive in their respective non-clinical habitats. In the event that the strains are transmitted into clinical settings, the antibiotic resistance genes encoded by the plasmids would perhaps be able to equip them with persistence against antimicrobial treatment. However, more work is required to prove this speculation.

## Conclusion

Eight complete plasmid sequences in *Pandoraea* were characterized and comparatively studied for the first time. These novel plasmids were successfully characterized according to the plasmid maintenance systems (replication, segregation, TA systems), conjugation, virulence and antibiotic resistance categories. Diverse and interesting features have been observed in the plasmids which include the possibility of host machinery interaction in plasmid segregation and replication. The characterization of the plasmid maintenance system according to replication, segregation and TA systems is important as we will not only gain insights into the mechanisms of plasmid survival in *Pandoraea*, but also perhaps aid in the treatment of *Pandoraea* pathogenicity. The mechanisms of plasmid maintenance can serve as targets in drug design, in order to prevent the continuous presence of virulent plasmids through cell generations. The knowledge of plasmid self-transmission through conjugation genes identification would be useful in the treatment of diseases especially when the self-transmitted plasmid itself carries virulence and antibiotic resistance genes. Other than being equipped with pathogenicity characteristics, plasmids with genes related to virulence and antibiotic resistance, provide the respective host strains with an advantage in adapting to their environment. Future studies should be carried out in confirming the functions of the predicted genes and discovering any hidden functional mechanisms.

## Author contributions

DY, KKT, WFY, and KGC carried out data collection and experiments. KGC conceived the idea and obtained funding. WFY handled the project finance. DY performed the analyses, interpretation of data and wrote the manuscript. All authors proofread and approved the final version of the manuscript.

### Conflict of interest statement

The authors declare that the research was conducted in the absence of any commercial or financial relationships that could be construed as a potential conflict of interest.

## Supplementary material

Supplementary Figure S1 **Plasmids found in *P. faecigallinarum* DSM 23572^T^: (A) pPF72-1 and (B) pPF72-1**. Genes located on the positive strand are drawn on the outside of the plasmid circle whereas genes located on the negative strand are drawn on the inside: conjugation genes (pink); replication genes (dark blue); partitioning genes (green); TA genes (red); virulence genes (yellow); antibiotic resistance genes (light blue); others (gray).

Supplementary Figure S2 **Plasmids found in *P. oxalativorans* DSM 23570^T^: (A) pPO70-1, (B) pPO70-2, (C) pPO70-3 and (D) pPO70-4**. Genes located on the positive strand are drawn on the outside of the plasmid circle whereas genes located on the negative strand are drawn on the inside: conjugation genes (pink); replication genes (dark blue); partitioning genes (green); TA genes (red); virulence genes (yellow); antibiotic resistance genes (light blue); others (gray).

Supplementary Figure S3 **Plasmid pPV15 from *P. vervacti* NS15**. Genes located on the positive strand are drawn on the outside of the plasmid circle whereas genes located on the negative strand are drawn on the inside: conjugation genes (pink); virulence genes (yellow); antibiotic resistance genes (light blue); others (gray).

Supplementary Figure S4 **Plasmid pPA35 from *P. apista* DSM 16535^T^**. Genes located on the positive strand are drawn on the outside of the plasmid circle whereas genes located on the negative strand are drawn on the inside: conjugation genes (pink); replication genes (dark blue); partitioning genes (green); TA genes (red); virulence genes (yellow); others (gray).

Supplementary Figure S5 **GC-skew plots: (A) pPF72-1; (B) pPF72-2**. The *oriV* is indicated by a minimum value of the cumulative GC-skew whereas *terV* is indicated by a maximum value of the cumulative GC-skew.

Supplementary Figure S6 **GC-skew plots: (A) pPO70-1; (B) pPO70-2; (C) pPO70-3; (D) pPO70-4**. The *oriV* is indicated by a minimum value of the cumulative GC-skew whereas *terV* is indicated by a maximum value of the cumulative GC-skew.

Supplementary Figure S7 **GC-skew plot: pPV15**. The *oriV* is indicated by a minimum value of the cumulative GC-skew whereas *terV* is indicated by a maximum value of the cumulative GC-skew.

Supplementary Figure S8 **GC-skew plot: pPA35**. The *oriV* is indicated by a minimum value of the cumulative GC-skew whereas *terV* is indicated by a maximum value of the cumulative GC-skew.

Supplementary Table S1 **Lists of putative protein-encoding genes**. Putative protein-encoding genes were highlighted according to replication (pink), segregation (green), conjugation (blue), ureolytic activity (yellow) and multidrug ABC transporter ATPase (orange) categories.

Supplementary Table S2 **List of putative toxin-antitoxin (TA) systems**.

Supplementary Table S3 **Conjugation information of additional genes in pPA35 and pPO70-4**. Yellow highlights indicate TA systems with toxin gene located upstream of the antitoxin gene.

Supplementary Table S4 **Lists of putative virulence genes**. Genes were highlighted according to the different functions: Type II secretion system (emerald), Type III secretion system (blue), Type IV secretion system (green), purine biosynthesis (peach), motility (yellow), iron acquisition (brown), cell surface (pink), DNA repair, replication and recombination (orange), adherence (gray), sialidase activity (red).

Supplementary Table S5 **List of putative resistance genes**. Genes were highlighted according to the different resistance functions: Multidrug ABC transporter ATPase (blue), tetracycline (green), beta-lactamase (pink), aminoglycoside (orange), fluoroquinolone (gray), macrolide (yellow), chloramphenicol (brown), lincosamide (emerald).
