# Supplementary material for: Characterization and Comparative Overview of Complete Sequences of the First Plasmids of Pandoraea across Clinical and Non-clinical Strains
Source: Front Microbiol. 2016 Oct 14;7:1606. doi: 10.3389/fmicb.2016.01606 (PMC5064223; doi:10.3389/fmicb.2016.01606)
Supplement: Supplementary Figure S1 — Plasmids found in P. faecigallinarum DSM 23572T: (A) pPF72-1 and (B) pPF72-1. Genes located on the positive strand are drawn on the outside of the plasmid circle whereas genes located on the negative strand are drawn on the inside: conjugation genes (pink); replication genes (dark blue); partitioning genes (green); TA genes (red); virulence genes (yellow); antibiotic resistance genes (light blue); others (gray). [file DataSheet1.pdf]

Supplementary Material

Characterization and Comparative Overview of Complete Sequences of the First Plasmids of *Pandoraea* across Clinical and Non-clinical Strains

Delicia Yong, Kok Keng Tee, Wai-Fong Yin, Kok-Gan Chan*

*** Correspondence:** Kok-Gan Chan: kokgan@um.edu.my

1 Supplementary Figures

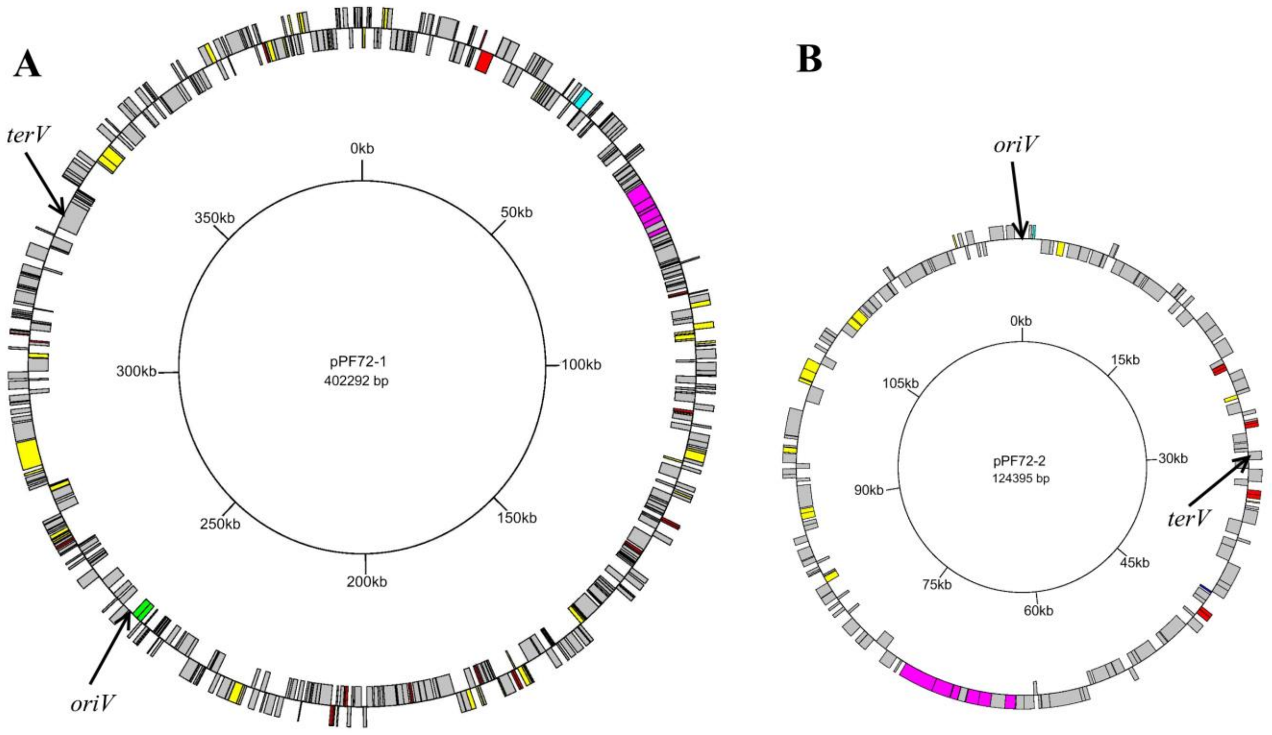

SUPPLEMENTARY FIGURE S1 | Plasmids found in *P. faecigallinarum* DSM 23572^{T}: (A) pPF72-1 and (B) pPF72-1. Genes located on the positive strand are drawn on the outside of the plasmid circle whereas genes located on the negative strand are drawn on the inside: conjugation genes (pink); replication genes (dark blue); partition genes (green); TA genes (red); virulence genes (yellow); antibiotic resistance genes (light blue); others (grey).

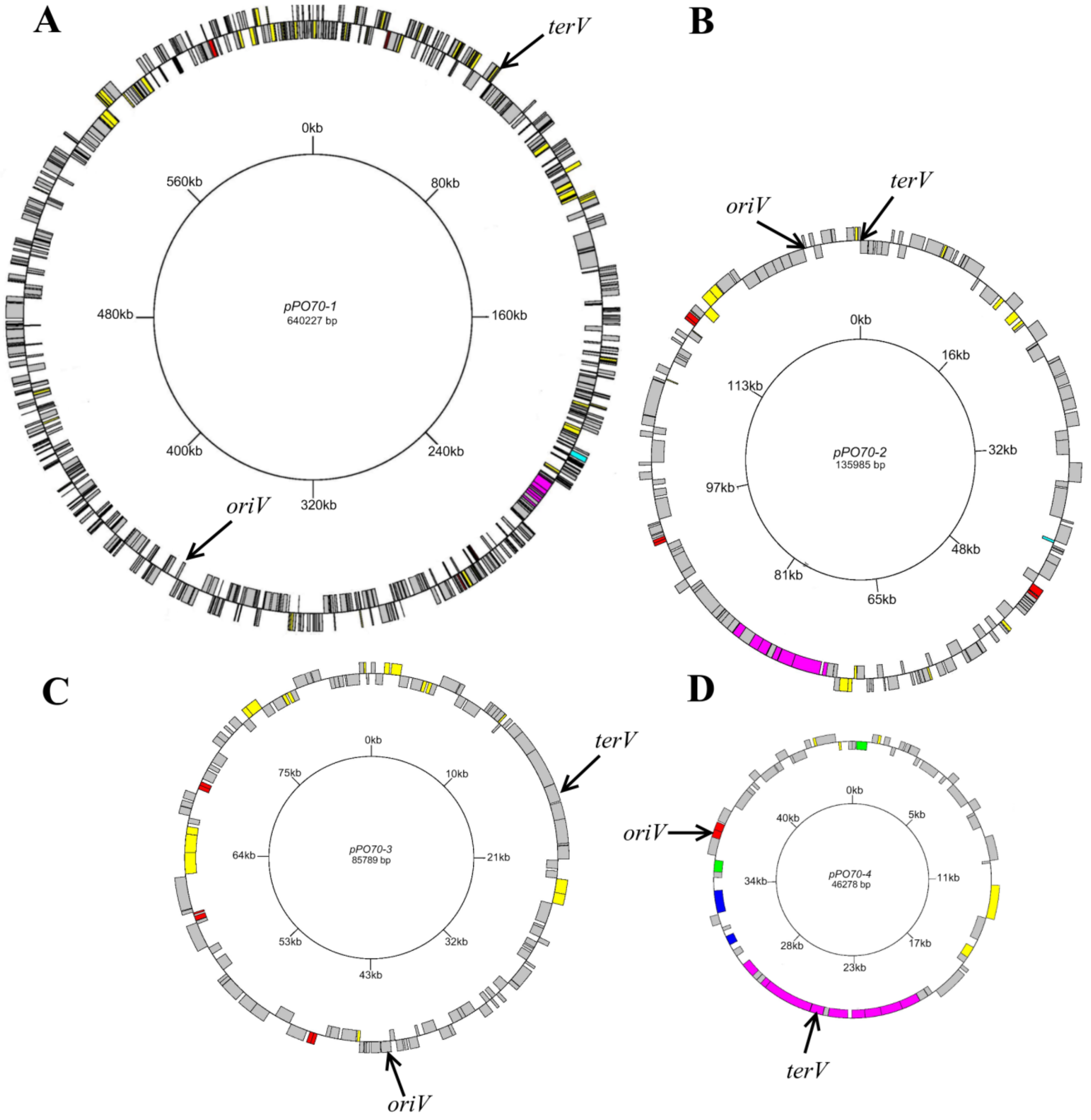

SUPPLEMENTARY FIGURE S2 | Plasmids found in *P. oxalativorans* DSM 23570^T: (A) pPO70-1, (B) pPO70-2, (C) pPO70-3 and (D) pPO70-4. Genes located on the positive strand are drawn on the outside of the plasmid circle whereas genes located on the negative strand are drawn on the inside: conjugation genes (pink); replication genes (dark blue); partition genes (green); TA genes (red); virulence genes (yellow); antibiotic resistance genes (light blue); others (grey).

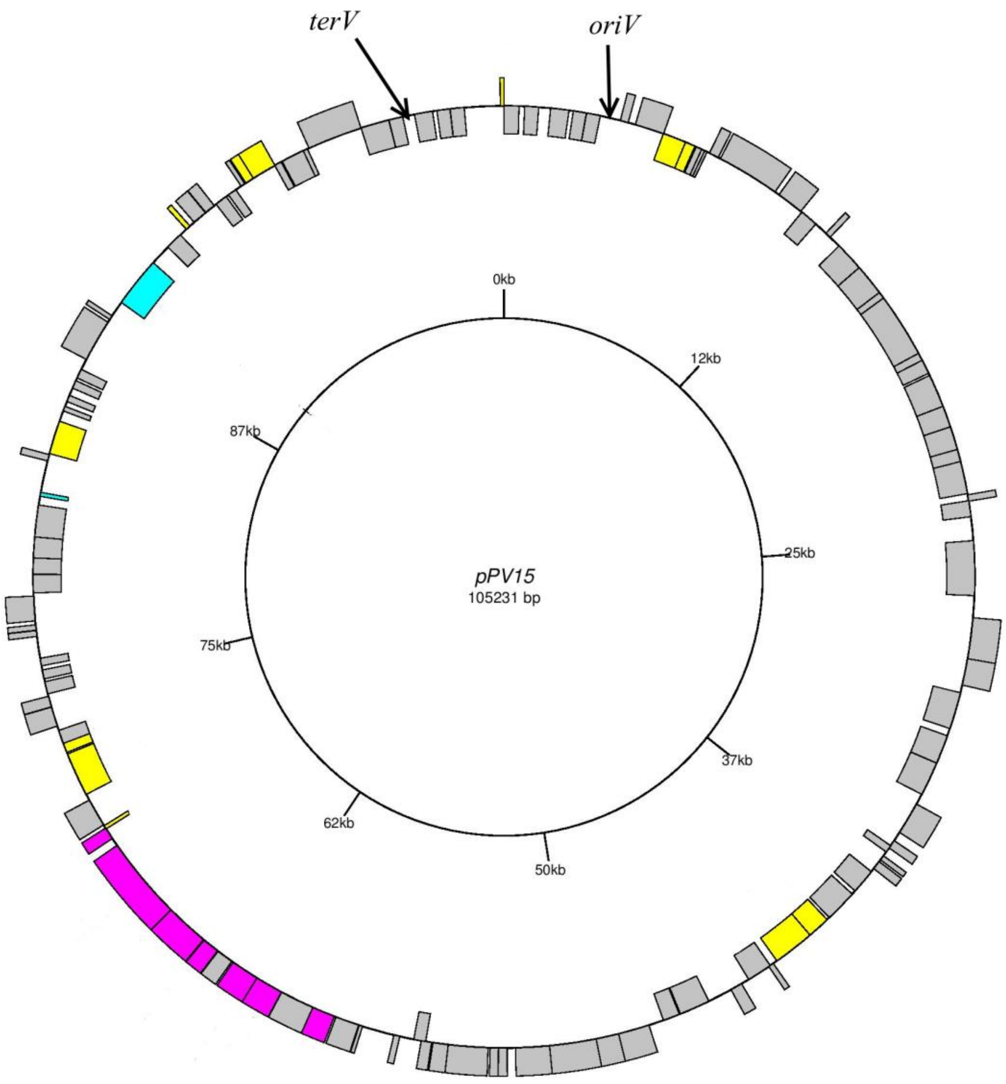

SUPPLEMENTARY FIGURE S3 | Plasmid pPV15 from *P. vervacti* NS15. Genes located on the positive strand are drawn on the outside of the plasmid circle whereas genes located on the negative strand are drawn on the inside: conjugation genes (pink); virulence genes (yellow); antibiotic resistance genes (light blue); others (grey).

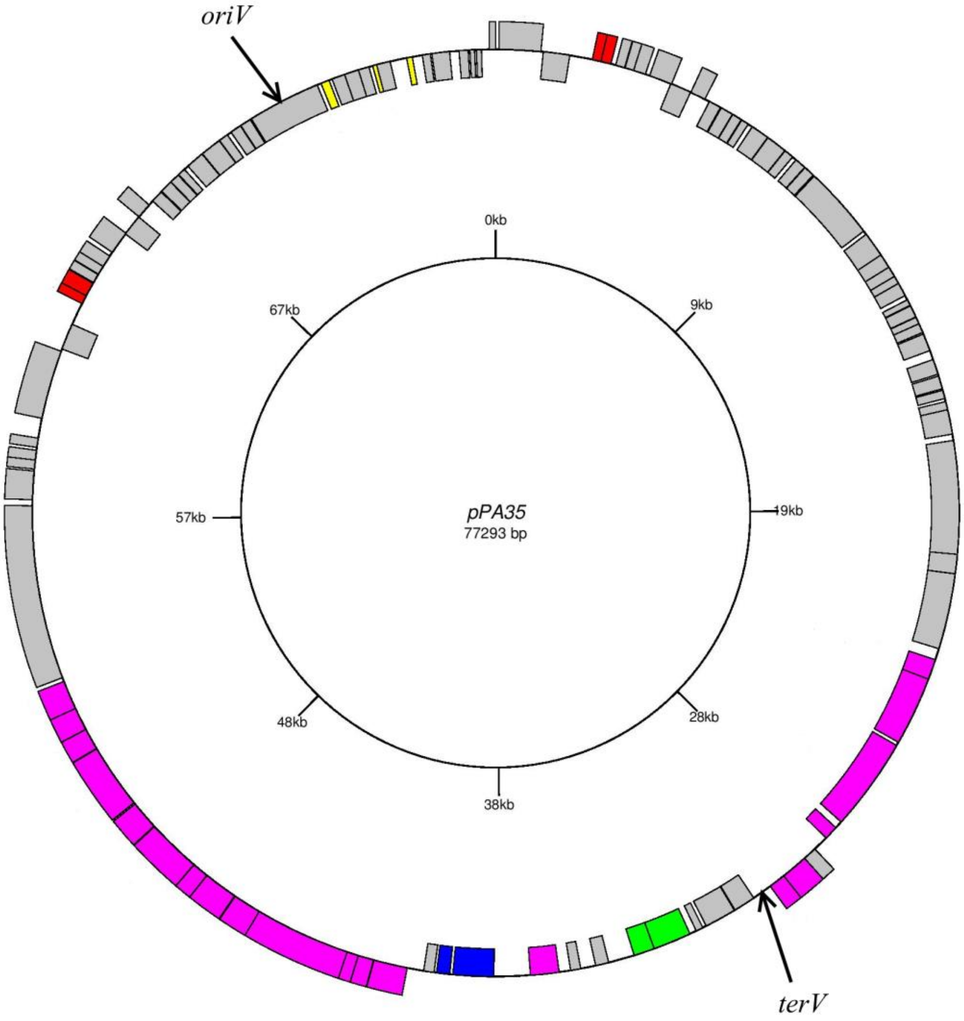

SUPPLEMENTARY FIGURE S4 | Plasmid pPA35 from *P. apista* DSM 16535^{T}. Genes located on the positive strand are drawn on the outside of the plasmid circle whereas genes located on the negative strand are drawn on the inside: conjugation genes (pink); replication genes (dark blue); partition genes (green); TA genes (red); virulence genes (yellow); others (grey).

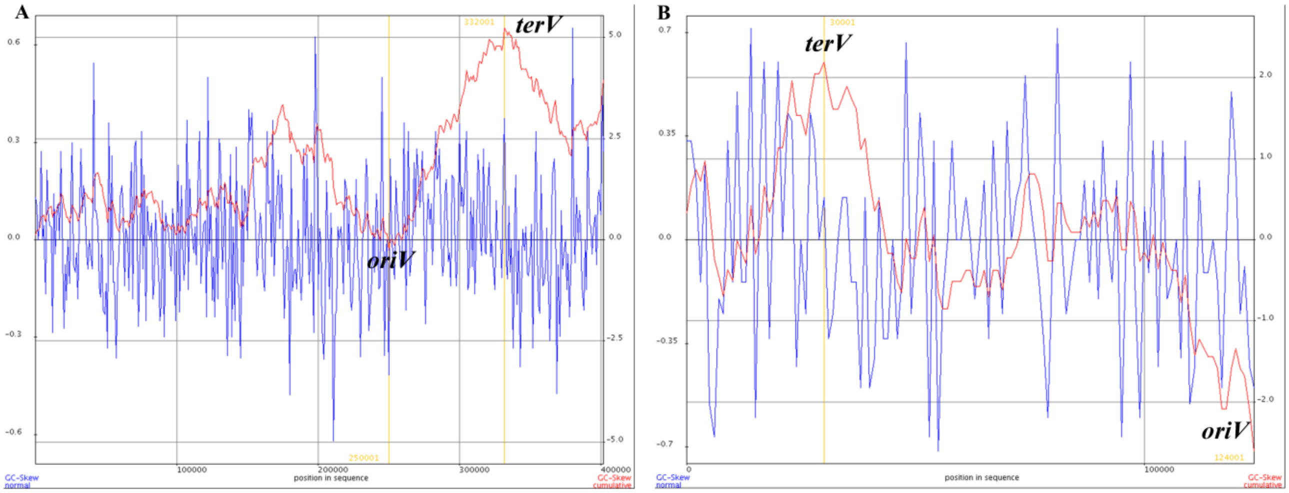

SUPPLEMENTARY FIGURE S5 | GC-skew plots: (A) pPF72-1; (B) pPF72-2. The *oriV* is indicated by a minimum value of the cumulative GC-skew whereas *terV* is indicated by a maximum value of the cumulative GC-skew.

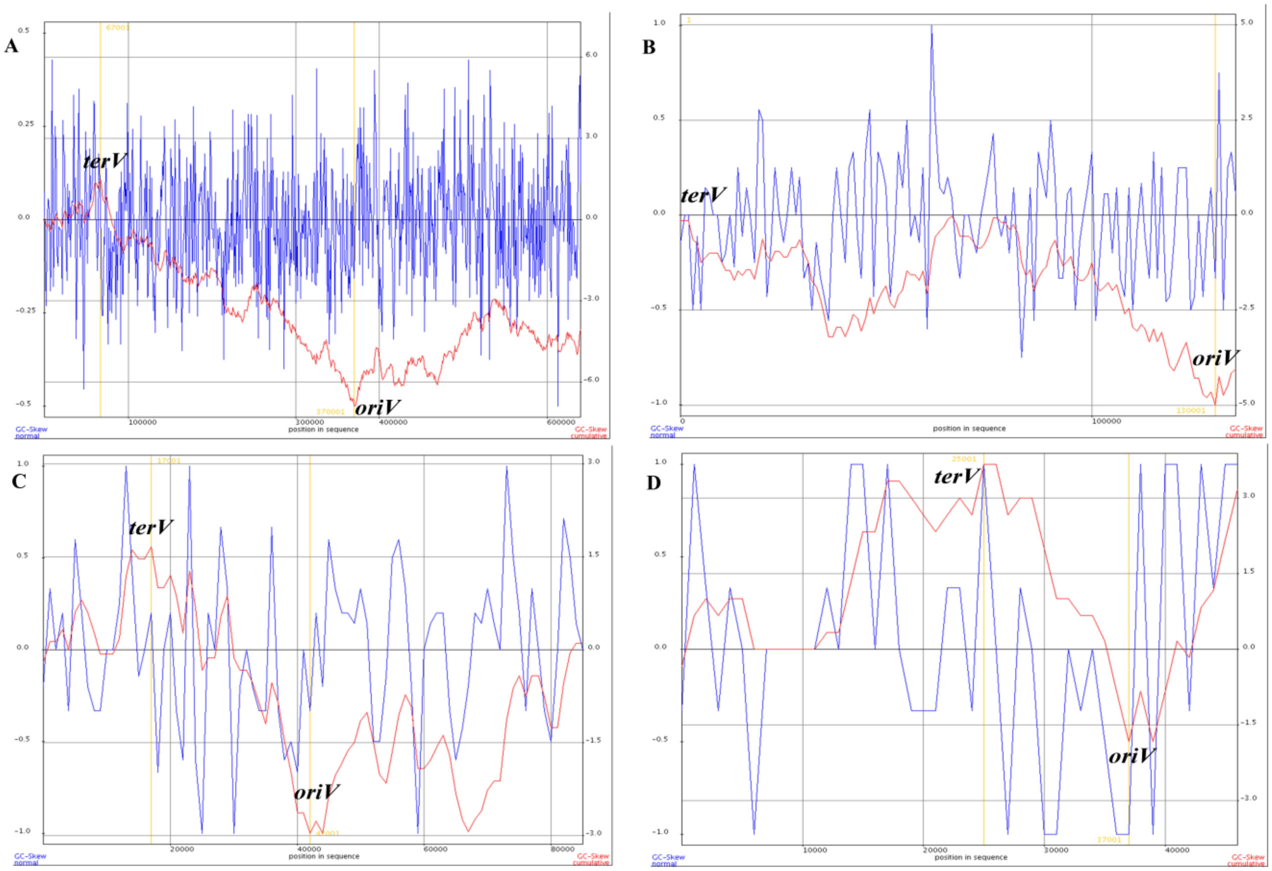

SUPPLEMENTARY FIGURE S6 | GC-skew plots: (A) pPO70-1; (B) pPO70-2; (C) pPO70-3; (D) pPO70-4. The *oriV* is indicated by a minimum value of the cumulative GC-skew whereas *terV* is indicated by a maximum value of the cumulative GC-skew.

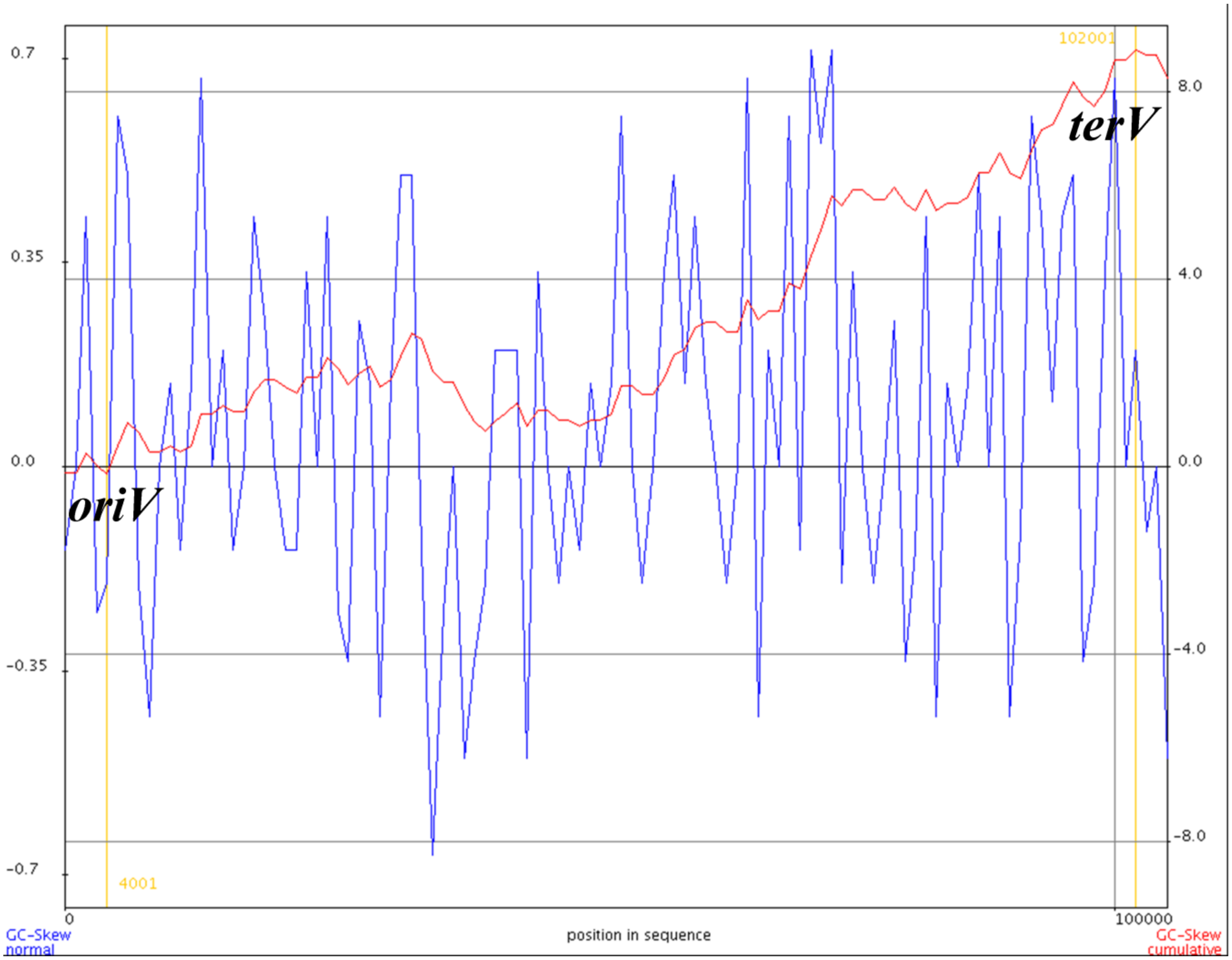

SUPPLEMENTARY FIGURE S7 | GC-skew plot: pPV15. The *oriV* is indicated by a minimum value of the cumulative GC-skew whereas *terV* is indicated by a maximum value of the cumulative GC-skew.

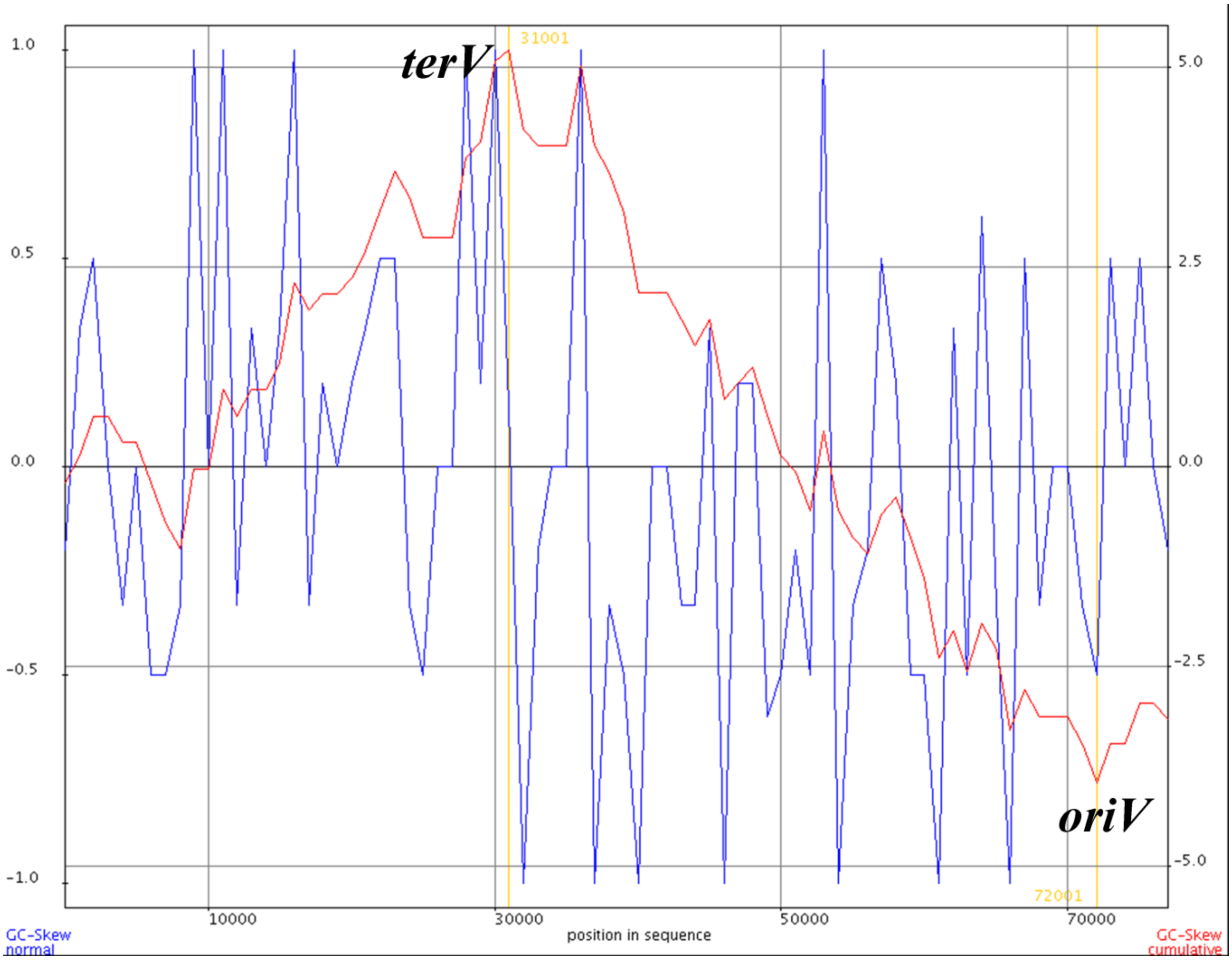

SUPPLEMENTARY FIGURE S8 | GC-skew plot: pPA35. The *oriV* is indicated by a minimum value of the cumulative GC-skew whereas *terV* is indicated by a maximum value of the cumulative GC-skew.
